# Influence of Fatty Liver on the Severity and Clinical Outcome in Acute Pancreatitis

**DOI:** 10.1371/journal.pone.0142278

**Published:** 2015-11-16

**Authors:** Chunfang Xu, Zhenguo Qiao, Yongda Lu, Deqing Zhang, Zhenyu Jia, Xiaohui Zhuang, Yuqi Shi, Ting Xu, Lihua Xing, Jiaqing Shen

**Affiliations:** 1 Department of Gastroenterology, The First Affiliated Hospital of Soochow University, Jiangsu, China; 2 Department of Gastroenterology, Affiliated Wujiang Hospital of Nantong University, Jiangsu, China; University of Szeged, HUNGARY

## Abstract

Acute pancreatitis (AP) is a common disease in the department of gastroenterology with variable severity, from being mild and self-limited to severe and fatal. The early diagnosis and accurate prediction of AP severity are of great importance. Our primary observation showed that fatty liver (FL) was frequently detected in patients with AP. In this retrospective study, we aimed to evaluate the relation between FL and the severity and outcomes of AP. The medical records of 2671 patients with AP were reviewed retrospectively, and characteristics of AP patients were recorded. FL was assessed by abdominal CT scan, and AP patients were categorized by the occurrence of FL for the analysis. The variation of mortality, clinical severity and the appearance of CT were analyzed between the non-FL group and FL groups. Compared with patients without FL, an obviously higher rate of death and higher frequency of severe AP (SAP) and necrotizing AP (ANP) were observed in patients with FL, as well as the incidence of local complications and systemic complications. Taking obesity into consideration, a higher rate of death and more severe AP were found in patients with FL, no matter whether they were obese or not. Alcoholic fatty liver (AFL) and non-alcoholic fatty liver (NAFL) were also separated for comparison in this study; the incidence of ANP and the clinical severity had no significant difference between the AFL and NAFL groups. In conclusion, FL could influence the severity and clinical outcome and may play a prognostic role in AP. This study is of clinical significance, because few reports have been previously issued on FL and AP.

## Introduction

Acute pancreatitis (AP) is a common disease in the department of gastroenterology with the variable severity, from being mild and self-limited to severe and fatal [1]. Severe AP (SAP) will occur in a certain percentage of patients, in which case, a localized inflammation with the emergence of the subsequent systemic inflammatory response secondary to the pancreatic damage. Ultimately responsible for the majority of the morbidity and mortality, the distant organs damage and the development of multiple organs dysfunction syndrome (MODS) are thought to be caused by the marked systemic inflammatory response [2, 3]. During the past few decades, the rate of death has not significantly declined, in spite of improvements in intensive care treatment [1, 4]. Therefore, the early diagnosis and accurate prediction of AP severity are still of great importance. Multifactorial scoring systems, like Ranson’s prognostic signs, the modified Glasgow, and the Acute Physiology and Chronic Health Evaluation II (APACHE II) scoring system, have been already eastablished and widely utilized, albeit with their own limitations. As a relatively simpler system, the Bedside Index for Severity of Acute Pancreatitis (BISAP) score has been recently developed and validated with reasonable diagnostic accuracy within 24 h after disease onset after AP [5, 6]. The presence of pancreatic necrosis is also believed to have a relationship with a worse prognosis in AP, which needs to be detected radiographically by dynamic intravenous contrast-enhanced computed tomography (CECT) of the abdomen. Now serum C-reactive protein (CRP) remains the most widely used individual biomarker, but the delay in peak levels makes it less useful on admission [7]. Therefore, it remains a difficult task to find a timely and effective method of the prognosis for AP, especially using clinical data which can be easily determined on admission [8, 9]. A large amount of researches have been carried on, with the identification of age and obesity as easily accessible and negative prognostic factors in AP [10–13]. But the studies are limited on the influence of fatty liver (FL) on the severity and clinical outcomes in AP, which is also commonly seen and is associated with several benign gastrointestinal and pancreato-biliary disorders [14].

Our primary observation showed that FL was frequently detected in patients with AP. In this retrospective study, we aimed to evaluate the relation between FL and the severity and outcomes of AP.

## Materials and Methods

This retrospective study was approved by Institutional review board of The First Affilated Hospital of Soochow University and written informed consent of each patient was obtained. Data of patients with AP, between January 2000 and June 2014, was searched by Haitai Database (Nanjing Haitai Information Technology Co., Ltd., Jiangsu, China). The study was conducted on patients with detailed demographic, clinical, and laboratory data, as well as the results of contrast-enhanced computed tomography (CECT). The diagnosis of AP required two of the following three features [15]: (1) abdominal pain consistent with AP (acute onset of a persistent, severe, epigastric pain often radiating to the back); (2) serum lipase activity or amylase activity at least three times greater than the upper limit of normal; and (3) characteristic findings of AP on contrast-enhanced computed tomography (CECT). The CECT was undergone around 7 days (5–10 days) after the onset of AP. Before hospital discharge, all MSAP and SAP cases were tested by ultrasound or CT scan to determine late-stage complications (including abscesses and pseudocysts). Exclusion criteria of this study included (1) chronic cardiac and pulmonay diseases; (2) previous history of pancreatic diseases, including acute pancreatitis, chronic pancreatitis and pancreatic cancer; (3) chronic renal failure; (4) chronic liver dysfunction; (5) a history of malignancy.

### Assessment of Fatty Liver

As previously reported, abdominal CT scan is a reliable noninvasive imaging method to detect hepatic steatosis, especially of moderate or greater severity [16]. The liver normally has a higher CT-attenuation value compared with the spleen, and the presence of FL will lead to a reversal of the liver/spleen attenuation ratio. By obtaining minimum, maximum, and mean Hounsfield units (HU) for regions of interest (ROI) > 100 mm^2^, the liver and the spleen attenuation values were measured, with two ROIs placed in the right liver lobe, one ROI in the left hepatic lobe, and one ROI in the spleen. The mean hepatic HU was derived by averaging the mean HU across all liver ROIs. The liver/spleen ratio was calculated by dividing the mean hepatic HU measurements by the mean spleen HU measurements. FL was defined as a liver/spleen ratio < 1.0 and classiefied into mild FL (liver/spleen ratio > 0.7 and < 1.0), moderate FL (liver/spleen ratio > 0.5 and ≤ 0.7), and severe FL (liver/spleen ratio ≤ 0.5). The readers were blinded to all clinical and demographic data.

### Definition of the Severity of Acute Pancreatitis

The severity of AP was defined, based on the 2012 revision of the Atlanta classification and definitions by international consensus [15]. AP was subdivided into two types: interstitial edematous pancreatitis (AEP) and necrotising pancreatitis (ANP), in accordance with the appearance of CECT and the existence of pancreatic necrosis [17]. This clinical classification defined three degrees of severity: mild AP (MAP), moderately severe AP (MSAP), and severe AP (SAP). MAP was defined as a diagnosed AP without organ failure and local or systemic complications. Diagnosed as MSAP, patients were accompanied with remote organ failure that resolves within 48 h, and/or local or systemic complications without persistent organ failure. Patients were classifed as SAP if remote organ failure was present for more than 48 h.

### Definition of Local Complications

Local complications were comprised of acute peripancreatic fluid collection, pancreatic pseudocyst, acute necrotic collection and walled-off necrosis. Other local complications of AP included gastric outlet dysfunction, splenic and portal vein thrombosis, and colonic necrosis [15].

### Definition of Organ Failure

Organ failure included at least one of the following features: (1) shock (systolic pressure <90 mmHg); (2) pulmonary insufficiency (PaO_2_ ≤60 mmHg); (3) renal failure (serum creatinine >2.0 mg/dL after hydration); (4) gastrointestinal bleeding (>500 ml/24 h); (5) disseminated intravascular coagulation (platelets ≤100,000/mm^3^, fibrinogen ≤100 mg/dL, fibrin split products >80 μg/mL); (6) a severe metabolic disturbance (serum calcium ≤7.5 mg/dL); (7) the presense of SIRS, which was defined by presence of two or more following criteria: (a) heart rate >90 beats/min, (b) core temperature <36°C or >38°C, (c) white blood count <4000 or >12000/mm^3^, (d) respirations >20/min [17].

### Definition of Obesity

BMI was calculated as weight in kilograms divided by height in meters squared (kg/m^2^). According to the World Health Organization (WHO) Western Pacific Region, obesity was classified by BMI, and BMI ≥25 kg/m^2^ defined an obese state [18].

### Definition of AFLD and NAFLD

Alcoholic fatty liver disease (AFLD) was diagnosed by the presence of following findings: (1) There was a history of alcoholic drink habit, and ethanol intake was more than 40g per day in men (20g per day in women) for more than 5 years; (2) hepatic steatosis was detected either by imaging or histology; (3) specific diseases that could lead to steatosis were excluded, such as viral hepatitis, drug-induced liver disease, autoimmune liver disease, and metabolic or hereditary liver disease [19]. Non-alcoholic fatty liver disease (NAFLD) was diagnosed by the presence of following findings: (1) steatosis was detected either by imaging or histology; (2) the alcoholic liver disease was excluded, and alcohol consumption was less than 140g per week in men (70g in women) in the past 12 months; (3) specific diseases that could lead to steatosis were excluded as mentioned above [20].

### Statistical Analysis

Continuous variables were expressed as means (± SD) and categorical variables as absolute or relative frequencies, and then analyzed using SPSS PC version 18.0 for Windows (SPSS Inc., Chicago, IL, USA). The criterion of statistical significance was p-values of <0.05. The significances of differences between the distributions of quantitative variables were assessed by using the Student’s *t*-test, and qualitative variables by using the chi-square test. Non-parametric data was assessed using the Whitney *U*-test. Relationships between FL and the severity of AP were assessed using odds ratios (ORs) with 95% confidence intervals (CIs).

## Results

2671 patients were finally enrolled in this study, and there were 1560 (58.41%) biliary pancreatitis patients, 592 (22.16%) alcohol-induced pancreatitis, 303 (11.34%) hyperlipidemic pancreatitis, and 216 (13.85%) with other etiologies, such as drugs and infections. According to the definition, there were 1911 (71.55%) MAP, 538 (20.14%) MSAP and 222 (8.31%) SAP among these patients, 68 (2.55%) of who finally died. The characteristics of AP patients at admission are summarized in Table 1. FL was found in 480 enrolled patients, and significant differences were observed between the non-FL group and FL group, in gender, etiology, BMI, the rate of obesity and the concentration of serum C-reactive protein (CRP) within 24 h after the onset of AP. The frequency of alcohol-induced pancreatitis and hyperlipidemic pancreatitis prevailed in patients with FL. The BMI value and the rate of obesity in patients with FL were also higher, as well as the serum CRP level. However, no significant differences were found in age, APACHE II score, incidence of diabetes and serum triglyceride level between FL group and non-FL group (Table 1). An obviously higher rate of death was observed in FL group (6.46%) than that in non-FL group (1.69%) (Odds ratio: 4.019, 95%CI: 2.467–6.547). The incidences of local complications and systemic complications were also higher in FL group (22.08% and 40.42%) than those in non-FL group (10.81% and 16.07%), as well as the incidence of SIRS (38.13% versus 17.89%). Metabolic deficiency and infection also developed more frequently in FL group (non-FL group: 9.86% and 37.70%; FL group: 29.58% and 46.46%) (Tables 2 and 3).

**Table 1:** Characteristics of enrolled AP patients.

	Non-FL (N = 2191)	FL (N = 480)	
Age (years)^a^	52.6 (20.78)	49.33 (13.06)	
Gender (M/F)	1268/923 (57.9%)	322/158 (67.08%)	*
Biliary cause (%)	1358 (61.98%)	202 (42.08%)	*
Alcohol cause (%)	450 (20.54%)	142 (29.58%)	
Hyperlipidemia cause (%)	202 (9.21%)	101 (21.04%)	
Other cause (%)	181 (8.26%)	35 (7.29%)	
BMI^b^	24.2 (20.5–28.1)	26.4 (24.1–29.6)	*
Obesity, n (%)	740 (33.77%)	237 (49.38%)	*
Diabetes, n (%)	128 (5.84%)	29 (6.04%)	
APACHE II score^b^	4.5 (2.0–7.5)	7.0 (4.0–12.5)	
Serum triglyceride level (mmol/L)^a^	2.98±1.92	4.73±2.05	
Serum C-reactive protein (mg/L)^a^	46.32±11.27	83.96±15.83	*

AP: acute pancreatitis; BMI: body mass index; APACHE: acute physiology and chronic health evaluation; FL: fatty liver.

^a^ Values expressed in mean (SD);

^b^ Values expressed in mean (range);

*p<0.05.

**Table 2:** Comparison of incidence of complications and outcomes in AP patients with FL or not.

	Non-FL (N = 2191)	FL (N = 480)	
Local complications, n (%)	237 (10.81%)	106 (22.08%)	*
Systemic complications, n (%)	352 (16.07%)	194 (40.42%)	*
Circulatory system, n (%)	52 (2.37%)	33 (6.88%)	*
Respiratory failure, n (%)	293 (13.37%)	165 (34.38%)	*
Renal failure, n (%)	97 (4.43%)	55 (11.46%)	*
SIRS, n (%)	392 (17.89%)	183 (38.13%)	*
Metabolic deficiency, n (%)	216 (9.86%)	142 (29.58%)	*
Infection, n (%)	826 (37.70%)	223 (46.46%)	*
Mortality, n (%)	37 (1.69%)	31 (6.46%)	*

AP: acute pancreatitis; FL: fatty liver; SIRS: systemic inflammatory response syndrome;

*p<0.05.

**Table 3:** Odds ratio for death, ANP and clinical severity in AP patients with FL.

	P value	Odds ratio	95%CI	
			Lower	Upper
Total				
Death	<0.01	4.019	2.467	6.547
ANP	<0.01	2.907	2.346	3.602
SAP	<0.01	2.635	1.958	3.546
MSAP+SAP	<0.01	2.644	2.156	3.242
Obese				
Death	<0.01	4.007	1.944	8.259
ANP	<0.01	3.169	2.297	4.372
SAP	<0.01	2.056	1.365	3.099
MSAP+SAP	<0.01	2.393	1.764	3.246
Non-obese				
Death	<0.01	3.796	1.925	7.484
ANP	<0.01	2.685	2.002	3.602
SAP	<0.01	3.132	2.051	4.782
MSAP+SAP	<0.01	2.377	1.803	3.134

ANP: acute necrotizing pancreatitis; AP: acute pancreatitis; FL: fatty liver; CI: confidence interval; SAP: severe acute pancreatitis; MSAP: moderately severe acute pancreatitis.

**Fig 1:**
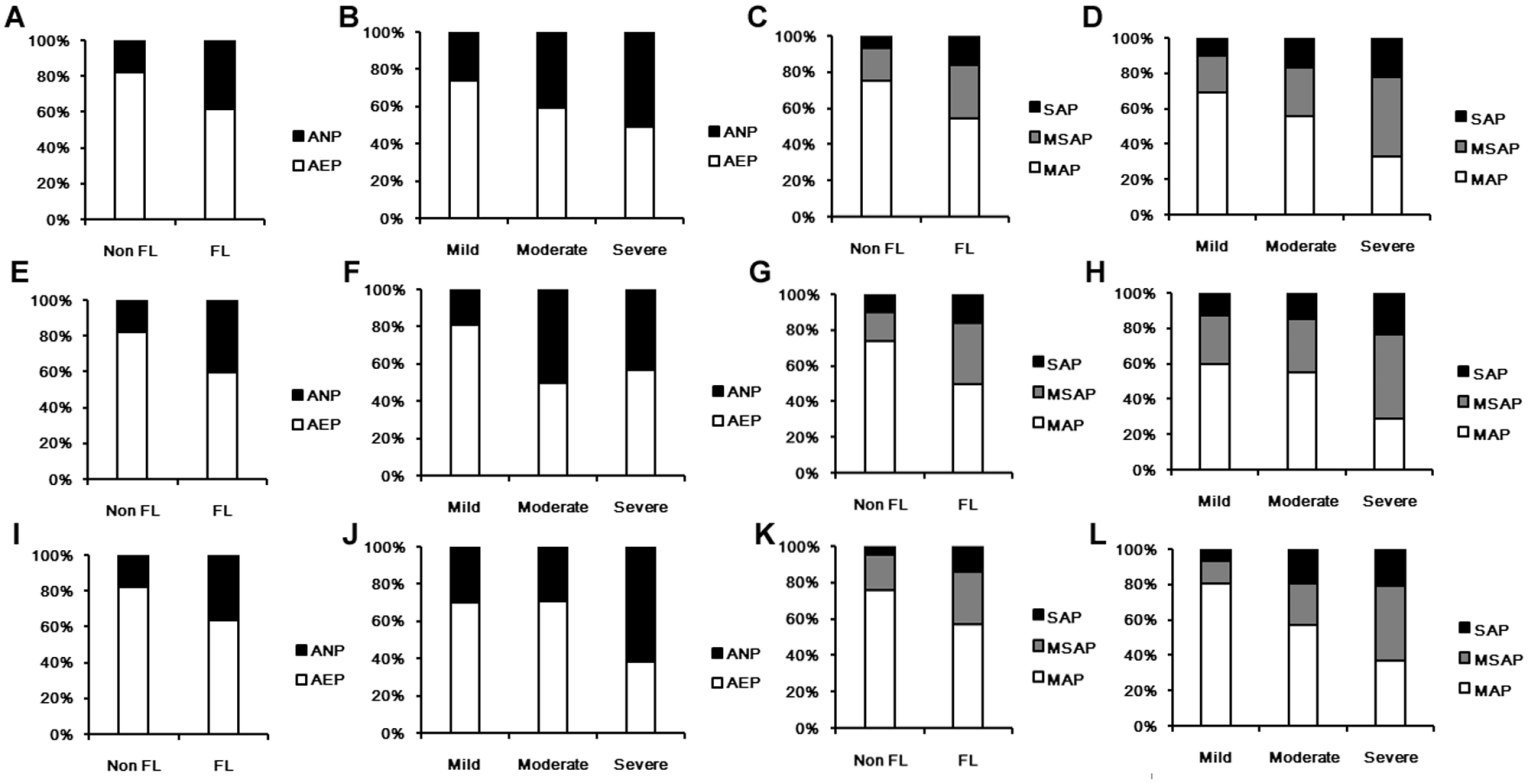
Comparison of the appearance of CECT and clinical severity of AP. (A, B) The incidence of ANP in total AP patients with or without FL; (C, D) The clinical severity of AP in total patients with or without FL; (E, F) The incidence of ANP in non-obese AP patients with or without FL; (G, H) The clinical severity of AP in non-obese patients with or without FL; (I, J) The incidence of ANP in obese AP patients with or without FL; (K, L) The clinical severity of AP in obese patients with or without FL; (B, D, F, H, J, L) FL patients were divided according to the severity of FL, including mild FL, moderate FL and severe FL. CECT: contrast-enhanced computed tomography; AP: acute pancreatitis; FL: fatty liver; AEP: acute edematous pancreatitis; ANP: acute necrotizing pancreatitis; MAP: mild acute pancreatitis; MSAP: moderately severe acute pancreatitis; SAP: severe acute pancreatitis.

According to the appearance of CECT, AP was divided into interstitial edematous pancreatitis (AEP) and necrotizing pancreatitis (ANP). The incidence of ANP in FL group (38.33%) was higher than that in non-FL group (17.62%) (OR: 2.907; 95%CI: 2.346–3.602). With the increase of the severity of FL, the incidence of ANP augmented in patients with FL (mild FL: 26.04%, moderate FL: 41.05% and severe FL: 51.24%). Based on the clinical classification of AP, the incidences of MSAP and SAP were still higher in FL group (30.42% and 15.83%) than those in non-FL group (17.89% and 6.66%). The odds ratio of SAP was 2.635 (95%CI: 1.958–3.546), and the odds ratio of MSAP+SAP was 2.644 (95%CI: 2.156–3.242). In patients with FL, the incidences of MSAP and SAP also raised along with the increase of severity of FL (mild FL: 20.71% and 11.24%, moderate FL: 27.37% and 16.84%, and severe FL: 48.76% and 22.31%) (Fig 1A–1D; Table 3).

**Fig 2:**
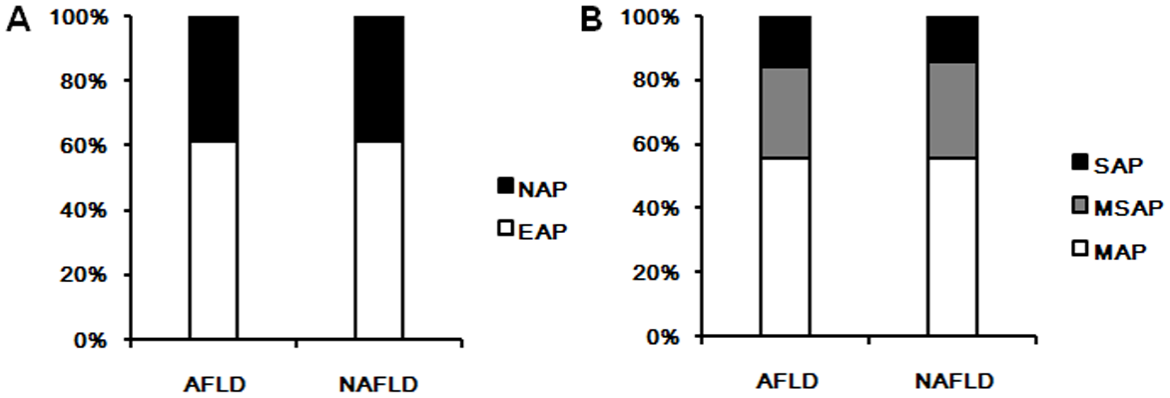
Comparison of the appearance of CECT and clinical severity of AP. (A) The incidence of ANP in AP patients with AFL or NAFL; (B) The clinical severity of AP in AP patients with AFL or NAFL. CECT: contrast-enhanced computed tomography; AP: acute pancreatitis; AFL: alcoholic fatty liver; NAFL: non- alcoholic fatty liver; AEP: acute edematous pancreatitis; ANP: acute necrotizing pancreatitis; MAP: mild acute pancreatitis; MSAP: moderately severe acute pancreatitis; SAP: severe acute pancreatitis.

When sub-classified the AP patients according to the occurrence of obesity, a worse prognosis was found both in obese patients and non-obese patients with FL. In non-obese patients, the rate of death in non-FL group was 1.59%, and 5.76% in FL group (OR: 3.796, 95%CI: 1.925–7.484). The incidence of ANP in FL group (36.63%) was more frequent than that in non-FL group (17.71%) (OR: 3.132, 95%CI: 2.051–4.782). The higher incidences of MSAP and SAP were again found in FL group (FL group: 29.63% and 14.81% versus non-FL group: 19.10% and 5.10%). The odds ratio of SAP was 3.132 (95%CI: 2.051–4.782), and MSAP+SAP was 2.377 (95%CI: 1.803–3.134) (Fig 1E–1H; Table 3). In obese patients, the rate of death in non-FL group was 1.89%, and 7.17% in FL group (OR: 4.007, 95%CI: 1.944–8.259). The incidence of ANP in FL group was 40.08%, and 17.43% in non-FL group (OR: 3.169, 95%CI: 2.297–4.372). Similarly, the higher incidences of MSAP and SAP were higher in FL group (33.84% and 16.35%) than those in non-FL group (15.97% and 10.00%). The odds ratio of SAP was 2.056 (95%CI: 1.365–3.099), and MSAP+SAP was 2.393 (95%CI: 1.764–3.246). In AP patients with FL, whether obese or not, the incidences of ANP and the clinical severity deteriorated, following the increase of the severity of FL (Fig 1I–1L; Table 3). Alcoholic fatty liver (AFL) and non-alcoholic fatty liver (NAFL) were also separated for comparison in this study. In AP patients with FL, 126 patients were diagnosed as AFL and 290 as NAFL, according to the criteria mentioned before. The incidence of ANP and the clinical severity had no significant difference between AFL and NAFL group (ANP: 38.89% versus 38.62%; MSAP: 28.57% versus 30.34%; SAP: 15.87% versus 14.14%) (Fig 2).

## Discussion

Most of patients with AP have a favorable prognosis, however a quick process can be observed in SAP, which is sometimes associated with a complicated clinical course and higher mortality [1, 3]. Therefore, the assessment of the disease severity in a timely and accurate manner is vital for initiating the appropriate supportive management, especially within the first 24 h after symptoms onset, which will lead to the improvement of prognosis and the reduction of mortality in SAP [4]. In the last few decades, many biomarkers, radiological and clinical scoring systems have been developed and validated to monitoring changes in AP patients. However, these have not been entirely successful. The Glasgow, Acute Physiology and Chronic Health Evaluation II (APACHE II), and Ranson scoring systems and plasma C-reactive protein (CRP) are still the most widely used parameters and form the part of many guidelines, though their use does come with its own limitations. An objective, accurate, fast and simple method is still necessary for the early intervention of potential SAP, particularly in an emergency room [21–23]. Age and obesity are confirmed as risk factors for a more severe form of AP, and can be measured at the very early stage. Yet, the researches on the prognostic role of FL in AP are relatively limited. Howbeit FL is associated with the pathogensis of numerous pancreato-biliary disorders [14].

Fatty liver disease (FLD) is thought to be a hepatic metabolic disorder, which is a condition where neutral fat accumulates in liver cells and may be accompanied by progressive inflammation of the liver [24]. In light of the contribution of alcohol, FL may be categorized as alcoholic liver disease (ALD) or non-alcoholic fatty liver disease (NAFLD), and the more severe forms of NAFLD as non-alcoholic steatohepatitis (NASH) [25]. With the prevalence of NAFLD is approximately 30% in developed countries and nearly 10% in developing countries, FL is a growing public health problem worldwide, which is increasingly recognized as an important cause of abnormal liver function tests [26]. The histological spectrum of ALD includes steatosis, hepatitis and fibrosis, and NAFLD can mimic the entire spectrum of hepatic changes in ALD, therefore, it is difficult to distinguish ALD from NAFLD histologically [27]. Current treatments for FL focus on the factors that may cause the disease, in general, including weight loss, cholesterol management, blood glucose control, or treatment of alcoholism [28]. FL can be detected by various imaging modalities. Previous reports show that ultrasonography, computed tomography (CT) and magnetic resonance imaging (MRI) have all demonstrated acceptable levels of sensitivity for detecting FL [29]. In our present study, CECT was utilized for the assessment of FL, because CECT was widely required for diagnosis and estimation of the severity in AP.

Limanond *et al* found a close correlation between the histological assessment of liver steatosis and the measurement of liver attenuation index (LAI), which was derived and defined as the difference between mean hepatic and mean splenic attenuation in CT scan [16]. Therefore, we used the difference of LAI to represent the severity of FL. In our study, the occurrence of FL was associated with higher mortality and higher incidences of local and systemic complications, indicating that FL might have a predictive value in AP. But the mechanism of the association between FL and the worse prognosis of AP was not well investigated in the current study. The explanations may be as follows: (1) Fatty liver is often accompanied with obesity, which would induce a long-term chronic inflammatory state that facilitates the expansion of inflammatory reaction in AP. Obesity is thought to be associated with an amplified systemic inflammatory response in AP and is a prognostic factor for mortality, local, systemic complications and severity in AP. Moreover, adipose tissue is confirmed to be involved in the progression from local pancreatic damage to systemic inflammation during AP by fat necrosis, the release of virulence factors and an excessive inflammatory reaction [30–32]. (2) Kuppfer cells in the hepatic tissues, whose capacity increases in FL, play an important role in the course of AP by releasing the inflammatory mediators, resulting in the exacerbation of systemic inflammatory response and pulmonary injury [33]. Several studies indicate the obesity is a prognostic factor of the development of local and systemic complications in AP, although the mortality among obese patients is only slightly higher, leading to a proposal that the APACHE II scoring system be modified to take obesity into account [34]. While, in our study, we found that FL was a risk factor for the deterioration of AP, no matter the obesity occurred or not. Therefore, it is also plausible to take FL into account in the AP severity scoring system, and more attention is needed to be paid to the AP patients with previous known FL at admission.

Alcoholic liver disease (ALD) represents a large proportion of chronic liver disease, and a considerable fraction of heavy drinkers develop severe alcoholic hepatitis (AH), which can complicate any stage of alcoholic liver injury [19, 35]. Along with this, alcohol abuse is also commonly associated with the development of both acute and chronic pancreatitis, and the cumulative effect of ethanol on the pancreas may predispose the pancreas to pancreatitis [36]. It is widely believed that ethanol and its metabolites sensitizes the pancreas to injury, by their deleterious effects on acinar cells properties, including calcium signaling, secretion of zymogens, autophagy, cellular regeneration, the unfolded protein response, and mitochondrial membrane integrity [37]. On the other hand, non-alcoholic fatty liver disease (NAFLD) is characterized by the accumulation of triglycerides in hepatocytes. NAFLD is frequently associated with obesity, type 2 diabetes mellitus, hyperlipidemia, and insulin resistance and is associated with increased cardiovascular- and liver-related mortality [38]. Though the molecular mechanism underlying NAFLD progression is not completely understood, its pathogenesis has often been interpreted by the "double-hit" hypothesis. The primary insult or the "first hit" includes lipid accumulation in the liver, followed by a "second hit" in which pro-inflammatory mediators induce inflammation, hepatocellular injury, and fibrosis [39]. While, some studies suggest that fatty acids and their metabolites may be the true lipotoxic agents that contribute to NAFLD progression [40, 41]. Meanwhile, hyperlipidemia is also a risk factor for both acute and chronic pancreatitis and the role of fatty acids in mediating damage has received increasing attention in recent years [42]. In our study, alcoholic fatty liver (AFL) and non-alcoholic fatty liver (NAFL) were both found in AP patients (126/2671 and 290/2671). Although the significant difference was found between non-FL group and FL group, the incidence of ANP and the clinical severity of had no significant difference between AFL and NAFL group, when FL patients were separated for subgroup analysis. High alcohol intake remains harmful since it is associated with elevated plasma triglycerides, but also with cardiovascular disease, alcoholic fatty liver disease and the development of pancreatitis. Alcohol-induced hypertriglyceridemia is due to increased very-low-density lipoprotein secretion, impaired lipolysis and increased free fatty acid fluxes from adipose tissue to the liver [43]. Therefore, alcohol and hyperlipiemia may have the common pathogenic mechanism in AP, which could explain the similarity of AP severity between AFL and NAFL.

As a retrospective study, the disadvantages of the study also exist. The CECT was undergone around 7 days (5–10 d) after the onset of AP, and the time interval would certainly influence on the diagnosis of FL, because of the process of the disease and the early treatment. CECT is a non-invasive method, and is not an absolutely accurate method for the diagnosis of FL. However, the biopsy is relatively unavailable, especially in AP patients. Thus, CECT was still applied in this study. A further prospective experiment is now on process for the further evaluation whether the occurrence of FL at admission is a risk factor of SAP.

In conclusion, this study shows that fatty liver portended a higher risk of SAP. In AP patients with fatty liver, the mortality was significantly higher than AP patients without fatty liver. Fatty liver also developed more local complications and systemic complications in AP. This study is of clinical significance, because few reports have been previously issued on fatty liver and acute pancreatitis.
